# Patterns of adaptive servo-ventilation settings in a real-life multicenter study: pay attention to volume! Adaptive servo-ventilation settings in real-life conditions

**DOI:** 10.1186/s12931-020-01509-7

**Published:** 2020-09-21

**Authors:** Dany Jaffuel, Claudio Rabec, Carole Philippe, Jean-Pierre Mallet, Marjolaine Georges, Stefania Redolfi, Alain Palot, Carey M. Suehs, Erika Nogue, Nicolas Molinari, Arnaud Bourdin

**Affiliations:** 1 Department of Respiratory Diseases, Univ Montpellier, CHU Montpellier, 371, Avenue Doyen Giraud, 34295 Montpellier Cedex 5, France; 2 PhyMedExp, Univ Montpellier, CNRS, INSERM, CHU Montpellier, Montpellier, France; 3 Pulmonary Department and Respiratory Critical Care Unit, University Hospital Dijon, Dijon, France; 4 Centre des pathologies du sommeil, Hôpital Universitaire de la Pitié Salpêtrière, AP-HP, Paris, France; 5 Clinique des Bronches, Allergies et du Sommeil, Assistance Publique Hôpitaux de Marseille, France et INSERM U1067, CNRS UMR 7333 Aix Marseille Université, 13015, Marseille, France, Hôpital Saint-Joseph, 26, boulevard de Louvain, 13285 Marseille, France; 6 Department of Medical Information, Univ Montpellier, CHU Montpellier, Montpellier, France; 7 Clinical Research and Epidemiology Unit (URCE), Univ Montpellier, CHU Montpellier, Montpellier, France; 8 IMAG, CNRS, Univ Montpellier, CHU Montpellier, Montpellier, France

**Keywords:** Adaptive servo-ventilation, Setting, Minute volume, Tidal volume, Pressure, Cluster, Cardiopathy, Sleep-disordered breathing

## Abstract

**Backgrounds:**

To explain the excess cardiovascular mortality observed in the SERVE-HF study, it was hypothesized that the high-pressure ASV default settings used lead to inappropriate ventilation, cascading negative consequences (i.e. not only pro-arrythmogenic effects through metabolic/electrolyte abnormalities, but also lower cardiac output). The aims of this study are: i) to describe ASV-settings for long-term ASV-populations in real-life conditions; ii) to describe the associated minute-ventilations (MV) and therapeutic pressures for servo-controlled-flow versus servo-controlled-volume devices (ASV-F Philips®-devices versus ASV-V ResMed®-devices).

**Methods:**

The OTRLASV-study is a cross-sectional, 5-centre study including patients who underwent ASV-treatment for at least 1 year. The eight participating clinicians were free to adjust ASV settings, which were compared among i) initial diagnosed sleep-disordered-breathing (SBD) groups (Obstructive-Sleep-Apnea (OSA), Central-Sleep-Apnea (CSA), Treatment-Emergent-Central-Sleep-Apnea (TECSA)), and ii) unsupervised groups (*k*-means clusters). To generate these clusters, baseline and follow-up variables were used (age, sex, body mass index (BMI), initial diagnosed Obstructive-Apnea-Index, initial diagnosed Central-Apnea-Index, Continuous-Positive-Airway-Pressure used before ASV treatment, presence of cardiopathy, and presence of a reduced left-ventricular-ejection-fraction (LVEF)). ASV-data were collected using the manufacturer’s software for 6 months.

**Results:**

One hundred seventy-seven patients (87.57% male) were analysed with a median (IQ_25–75_) initial Apnea-Hypopnea-Index of 50 (38–62)/h, an ASV-treatment duration of 2.88 (1.76–4.96) years, 61.58% treated with an ASV-V. SDB groups did not differ in ASV settings, MV or therapeutic pressures. In contrast, the five generated *k*-means clusters did (generally described as follows: (C1) male-TECSA-cardiopathy, (C2) male-mostly-CSA-cardiopathy, (C3) male-mostly-TECSA-no cardiopathy, (C4) female-mostly-elevated BMI-TECSA-cardiopathy, (C5) male-mostly-OSA-low-LVEF). Of note, the male-mostly-OSA-low-LVEF-cluster-5 had significantly lower fixed end-expiratory-airway-pressure (EPAP) settings versus C1 (*p* = 0.029) and C4 (*p* = 0.007). Auto-EPAP usage was higher in the male-mostly-TECSA-no cardiopathy-cluster-3 versus C1 (*p* = 0.006) and C2 (*p* < 0.001). MV differences between ASV-F (*p* = 0.002) and ASV-V (*p* < 0.001) were not homogenously distributed across clusters, suggesting specific cluster and ASV-algorithm interactions. Individual ASV-data suggest that the hyperventilation risk is not related to the cluster nor the ASV-monitoring type.

**Conclusions:**

Real-life ASV settings are associated with combinations of baseline and follow-up variables wherein cardiological variables remain clinically meaningful. At the patient level, a hyperventilation risk exists regardless of cluster or ASV-monitoring type, spotlighting a future role of MV-telemonitoring in the interest of patient-safety.

**Trial registration:**

The OTRLASV study was registered on ClinicalTrials.gov (Identifier: NCT02429986). 1 April 2015.

## Introduction

Adaptive Servo-Ventilation (ASV) is a non-invasive ventilatory therapy that provides positive expiratory airway pressure and inspiratory pressure support based on servo-controlled-flow or -volume monitoring [1–4]. At the beginning of the 2000s, ASV was mainly developed for the treatment of central sleep apnea (CSA) associated with Chronic Heart Failure (CHF) and reduced Left Ventricular Ejection Fraction (LVEF, i.e. LVEF ≤45%) [5]. Unlike preliminary data demonstating short term benefits in terms of symptoms and physiology [6], the randomized SERVE-HF study reported an unexpected increase in cardiovascular mortality with ASV-treatment [7]. To explain these conflicting results, it was hypothesized that the high-pressure ASV default settings used in the SERVE-HF study could lead to an inappropriate ventilation with cascading negative consequences (i.e. not only pro-arrythmogenic effects through alkalosis and hypocapnia related to hyperventilation, but also a direct lower cardiac output through ASV-pressurization) [8–10]. In these CHF patients with reduced LVEF, the detrimental cardiovascular effects of alkalosis and hypocapnia consecutive to the ASV related hyperventilation remains theoretical. Indeed, no published study describing simultaneously measured ASV minute-volume (MV) and physiopathological data currently exists, despite a related passionate debate [11–14]. However, the potential negative effects of positive airway pressure support on cardiac output have been clearly demonstrated [15–19]. In this context, a specific warning against the use of high expiratory positive airway pressure (EPAP) in reduced LVEF patients exists [20]. Simultaneously, the auto-EPAP modes developed by ASV-manufacturers are available in daily practice, but lack an evidence base for superiority on fixed-EPAP in clinical and bench studies [2, 21], and consensual recommendations are clearly absent. The potential for cardiac output worsening due to specific pressure levels is not always counterbalanced by the neurovegetative response [19, 20, 22] and at-risk patients are likely those with a low pulmonary capillary wedge pressure [18] and a right ventricular dysfunction [19].

Notwithstanding the physiopathologic reasons for the observed cardiovascular mortality in the SERVE-HF study, whether the SERVE-HF results (a study performed with a volume monitored ASV (ASV-V)), can be extrapolated to non-SERVE-HF patients and/or patients treated with a flow monitored ASV (ASV-F) remains unknown. This is all the more important since the SERVE-HF “phenotype” (i.e. patients with CSA and a LVEF ≤45%) represents only 5.8 to 13.5% of the ASV-treated population [23–26].

Paradoxically, real-life data describing ASV-settings and their associated flow- versus volume- controlled monitoring are sparse [27, 28], and current recommendations [29, 30] do not mention ASV-setting guidelines of any kind. As a consequence, ASV-settings are empirical in nature and left to each physician’s discretion. In particular, how two important factors, (i) patient aetiologies/comorbidities and (ii) servo-monitoring type, combine and simultaneously impact ASV-settings is unknown and therefore, ASV settings remain expertise-dependent.

OTRLASV (Observational Transversal Real-life Study of ASV) is a multicentric cross-sectional study describing a cohort of patients who have undergone ASV for at least 1 year in real-life conditions. In a previous publication [24], we described the clinical characteristics and cardiological/pulmonary monitoring of these patients. With the objective of filling the literature gap left by the under-reporting of ASV-settings, the primary objective of the present paper is to describe the latter according to aetiologies/comorbidities. Secondarily, we describe and compare the related MV and therapeutic pressures for flow- versus volume-monitored ASV to explore a potential ASV-monitoring type effect.

## Methods

### Study design

The OTRLSAV study is an observational, cross-sectional, five-expert-centre study conducted on an exhaustive cohort of consecutive patients treated for at least 1 year with ASV for sleep apnea (ClinicalTrials.gov Identifier: NCT02429986). The protocol complied with the Declaration of Helsinki and was reviewed and approved by an independent ethics committee (*Comité de Protection des Personnes “Sud Méditérannée III”*; reference number 2014.11.04).

Detailed data about the global study design, procedures, demographic or sleep characteristics have been previously published [24]. The complementary ASV-setting analyses reported here focus on the unpublished ASV-treatment modalities of the 177-patient population. There were no ASV-setting recommendations established among the 5 centres and the 8 participating clinicians were free to choose ASV-brands and adjust ASV-settings as they saw fit.

### Study population

The study flow chart is depicted in Additional file 1. Two analyses of ASV settings and software-measured data were performed. Considering that initial diagnosis may impact ASV-settings and in line with our previous report [24], the first analysis was based on the initial diagnosed sleep-disordered breathing (SBD) groups (i.e., Central-Sleep-Apnea (CSA), Obstructive-Sleep-Apnea (OSA), and Treatment-Emergent-Central-Sleep-Apnea (TECSA) groups). How SDB groups were determined is detailed in Additional file 2 and previous reports [24, 25]. Because our previous publication demonstrated that ASV-settings could be modified over time consecutive to polygraphy/oxymetry monitoring [24], the second analysis was made on unsupervised groups created via a clustering algorithm combining baseline and follow-up variables (further details are given in the statistics section).

### Clinical data

The clinical information collected for the analysis included age, sex, anthropometry, the apnoea-hyponoea index (AHI; determined by initial polysomnography (PSG) or respiratory polygraphy (PG)), and the Epworth Sleepiness Scale (ESS). The presence/absence of (i) a continuous positive airway pressure (CPAP) trial prior to ASV initiation, (ii) cardiomyopathy and (iii) an altered LVEF were also noted.

### Device-collected data

Included patients were treated either by an ASV-F device (the BiPAP autoSV Advanced Sytem One or the BiPAP auto SV Advanced Dreamstation (Philips Respironics®, Murrysville, PA, USA)), or an ASV-V device (the Resmed AirCurve 10 CS PaceWave or the ResMed S9 AutoSet CS (a device without auto-adjusting expiratory positive airway pressure (EPAP), which is similar to the S9 VPAP Adapt used in ASV mode with fixed EPAP, historically marketed in the United States), Resmed®, Sydney, Australia)).

ASV data were collected using the manufacturer’s software. Data downloads were performed for the 6 months preceding the inclusion date regardless of the ASV-initiation date. Settings were detailed as follows: expiratory positive airway pressure (EPAP; fixed or minimum/maximum), inspiratory pressure support (IPS, minimum and maximum), maximum pressure, fixed backup respiratory rate (RR) or auto backup RR, and for the Philips Respironics device, the inspiratory support minimum pressurization time and slope level. The associated device-reported outcomes were summarized as follows: usage reported as the average hours/night for 6 months, residual AHI (AHI_flow_), a centrality measure for residual leaks (mean percentage of important leaks for Philips Respironics® devices, median unintentional leaks for Resmed® devices), mean/median RR, mean/median minute-ventilation (MV), and therapeutic pressures. In addition, we collected the interface type, and the presence of a heated humidifier/breathing tube.

Tidal volumes were obtained by dividing the measured volume-minute by device-RR. The theoretical tidal volume was calculated using an 8 ml/kg of ideal weight formula. Ideal weights were calculated according to the Lorentz equation [31].

### Statistical analyses

Continuous data were expressed as medians and interquartile ranges (IQ_25–75_). Qualitative parameters were expressed as numbers and percentages. Group comparisons were performed using ANOVA or Kruskal-Wallis tests for quantitative data. Qualitative variables were compared using Chi-square or Fisher exact tests. In case of a significant global effect, pairwise comparisons were performed using Holm corrections for multiple comparisons. A bilateral *p* value of < 0.05 was considered as indicating statistical significance. Data were pooled for ASV-setting variables common to both manufacturers (EPAP (fixed or minimum/maximum); IPS (minimum and maximum), maximum pressure, RR or auto RR). Otherwise, for IPS slope level and minimum inspiratory time, the data concerned only Philips Respironics devices.

Clustering methods were performed on standardized data after mean-imputation of missing values. Ascending hierarchical classification (AHC) using Ward’s method was used to determine the optimal number of clusters (*k)*. Subsequently, *k*-means clustering, initialized from the barycentres of the AHC partition, was performed to divide the population into *k* homogeneous groups. The variables used to establish clusters were: age, sex, body mass index (BMI), initial PG/PSG Obstructive Apnea Index (OAI/h), initial PG/PSG Central Apnea Index (CAI/h), CPAP previously used before ASV treatment, presence of cardiopathy, and presence of reduced LVEF.

All analyses were conducted by the Department of Research and Medical Information at the Montpellier University Hospitals using statistical software (SAS Enterprise Guide, version 7.3; SAS Institute; Cary, North Carolina, USA).

## Results

The 177 patients (87.6% male) analysed had a median age of 71 (IQ25–75: 65–77) years, a median body mass index of 29.9 (26.6–34.0) kg/m^2^, and a median initial AHI of 50/h (38–62). Sixty-eight patients (38.42%) were treated with ASV-F devices, and 109 (61.58%) via ASV-V devices. The median duration of ASV treatment was 2.88 years (1.76–4.96).

### SDB group comparisons

Table 1 summarises ASV-settings data for the SDB groups (CSA, OSA, and TECSA groups). No significant differences were found between SDB groups except for the presence/absence of heated breathing tube usage, which was more prevalent in the OSA group than in the CSA group. Of note, auto-EPAP usage was similar between the SDB groups (*p* = 0.369).

**Table 1:** Adaptive servo-ventilation settings for the OTRLASV population based on initial sleep-disordered-breathing diagnostic groups

	Total ***n*** = 177 (100%)	CSA group ***n*** = 105 (59.3%)	OSA group ***n*** = 36 (20.3%)	TECSA group ***n*** = 36 (20.3%)	***P***
**Fixed EPAP** (cmH_2_O)^b^	*n* = 121	*n* = 76	*n* = 22	*n* = 23	
	6.00	5.50	6.00	7.00	0.458
	[5.00–9.00]	[5.00–9.00]	[5.00–8.00]	[5.00–10.00]	
	(4.00–14.00)	(4.00–14.00)	(4.00–12.00)	(4.00–14.00)	
**Auto-EPAP** (cmH_2_O)^b^	*n* = 56	*n* = 29	*n* = 14	*n* = 13	
**EPAPmin**	4.00	4.00	5.00	5.00	0.143
	[4.00–5.50]	[5.00–5.00]	[4.00–6.00]	[4.00–5.00]	
	(4.00–13.00)	(4.00–13.00)	(4.00–8.00)	(4.00–10.00)	
**EPAPmax**	10	10	10	9	0.176
	[8.00–12.00]	[8.00–10.00]	[10.00–12.00]	[7.00–12.00]	
	(3.00–18.00)	(5.00–15.00)	(5.00–15.00)	(3.00–18.00)	
**IPS** (cmH_2_O)^b^	*n* = 176	*n* = 104	*n* = 36	*n* = 36	
**IPSmin**	3.00	3.00	3.00	3.00	0.284
	[0.00–3.00]	[3.00–3.00]	[0.00–5.00]	[0.00–3.00]	
	(0.00–15.00)	(0.00–15.00)	(0.00–13.00)	(0.00–7.50)	
**IPSmax**	10	10	10	10	0.400
	[9.00–10.00]	[10.00–10.00]	[8.00–11.00]	[8.50–10.00]	
	(3.00–20.00)	(6.00–20.00)	(3.00–17.00)	(5.00–15.00)	
**Pmax** (cmH_2_O)^b^	*n* = 129	*n* = 76	*n* = 27	*n* = 26	
	19	19	17	19.5	0.317
	[16.00–21.00]	[16.00–21.00]	[14.00–20.00]	[18.00–22.00]	
	(11.00–25.00)	(12.00–25.00)	(12.00–25.00)	(11–25.00)	
**Fixed backup RR**^b^ (cycle/min)	*n* = 39	*n* = 25	*n* = 8	*n* = 6	
	12	12	11.5	11.5	0.458
	[11.00–12.00]	[12.00–12.00]	[10.00–12.50]	[10.00–12.00]	
	(8.00–16.00)	(8.00–14.00)	(10.00–16.00)	(10.00–22.00)	
**Auto backup RR**^b^	*n* = 174	*n* = 104	*n* = 35	*n* = 35	0.697
	135 (77.59%)	79 (75.96%)	27 (77.14%)	29 (82.86%)	
**Slope level**^**c**^	*n* = 42	*n* = 24	*n* = 9	*n* = 9	
	2	2	2	2	0.951
	[2.00–3.00]	[2.00–3.00]	[2.00–3.00]	[2.00–3.00]	
	(1.00–6.00)	(1.00–6.00)	(1.00–3.00)	(1.00–3.00)	
**Ti min**^**c**^ (second)	*n* = 38	*n* = 25	*n* = 7	*n* = 6	
	1.65	1.70	1.70	1.70	0.929
	[1.40–1.80]	[1.40–1.80]	[1.50–1.80]	[1.20–1.80]	
	(1.00–2.20)	(1.00–2.20)	(1.00–2.00)	(1.20–2.10)	
**Interface**	*n* = 177	*n* = 105	*n* = 36	*n* = 36	
Oronasal	89 (50.28%)	53 (50.48%)	13 (36.11%)	23 (63.89%)	0.062
Nasal	81 (45.76%)	49 (46.67%)	20 (55.56%)	12 (33.33%)	0.160
Nasal Pillows	9 (5.08%)	4 (3.81%)	4 (11.11%)	1 (2.78%)	0.254
**Heated humidifier**	*n* = 177	*n* = 105	*n* = 36	*n* = 36	
	127 (71.75%)	70 (66.67%)	27 (75.00%)	30 (83.33%)	0.142
**Heated breathing tube**	*n* = 177	*n* = 105	*n* = 36	*n* = 36	
	47 (26.55%)	20 (19.05%)^a^	18 (50.00%)^a^	9 (25.00%)	0.001

Quantitative variables were summarized using medians, [IQ_25_–_75_] and (min – max), while categories were described by numbers and (%). The total percentage for interfaces is > 100% because multiple interfaces were used by 2 patients

Significant pairwise comparisons with Holm corrections are presented ^a^ for CSA vs. OSA groups

*CSA* Central sleep apnea, *EPAP* Expiratory positive airway pressure, *IPS* Inspiratory pressure support, *max* maximum, *min* minimum, *OSA* Obstructive sleep apnea, *Pmax* maximum pressure, *RR* Respiratory rate, *TECSA* Treatment emergent central sleep apnea, *Ti min* Inspiratory support minimum pressurization time

^b^For ASV-setting variables shared by both manufacturers, the data were pooled (EPAP (fixed or minimum/maximum); IPS (minimum and maximum), Pmax, RR or auto RR)

^c^For slope level and minimum inspiratory time, the data concern only Philips Respironics devices

Table 2 summarizes ASV-software MV data according to manufacturers and Additional file 3 reports ASV-software measured data. For a given manufacturer, no significant SDB group differences were found for MV or therapeutic pressures.

**Table 2:** Minute-ventilation mean/medians for adaptive servo-ventilation treatment according to initial sleep-disordered-breathing diagnostic groups and device types

	Total ***n*** = 177 (100%)	CSA group ***n*** = 105 (59.3%)	OSA group ***n*** = 36 (20.3%)	TECSA group ***n*** = 36 (20.3%)	***P***
**Philips Respironics®**	*n* = 68	*n* = 36	*n* = 18	*n* = 14	0.156
Mean MV (l/min)*	7.95	7.85	8.20	7.85	
*ASV flow-monitored devices.*	[7.20–9.30]	[7.20–8.95]	[7.50–10.40]	[6.20–9.60]	
	(4.90–14.80)	(4.90–11.70)	(5.80–14.80)	(5.10–11.20)	
**ResMed®**	*n* = 107	*n* = 68	*n* = 18	*n* = 21	0.258
Median MV (l/min)*	6.90	7.00	6.50	6.60	
*ASV volume-monitored devices.*	[6.00–7.80]	[6.30–7.75]	[5.80–7.50]	[5.90–8.50]	
	(2.50–11.40)	(3.60–11.40)	(2.50–10.10)	(3.80–10.40)	

Variables were summarized using medians, [IQ_25_–_75_] and (min – max)

*Note that for Philips Respironics® devices, the minute-ventilation is expressed as “mean” in the manufacturer software whereas for ResMed® devices, the minute-ventilation is expressed as “median” (thus preventing direct comparisons between these device types)

*ASV* Adaptive servo-ventilation, *CSA* Central sleep apnea, *MV* Minute-ventilation, *OSA* Obstructive sleep apnea, *TECSA* treatment emergent central sleep apnea

### K-means cluster comparisons

**Fig. 1:**
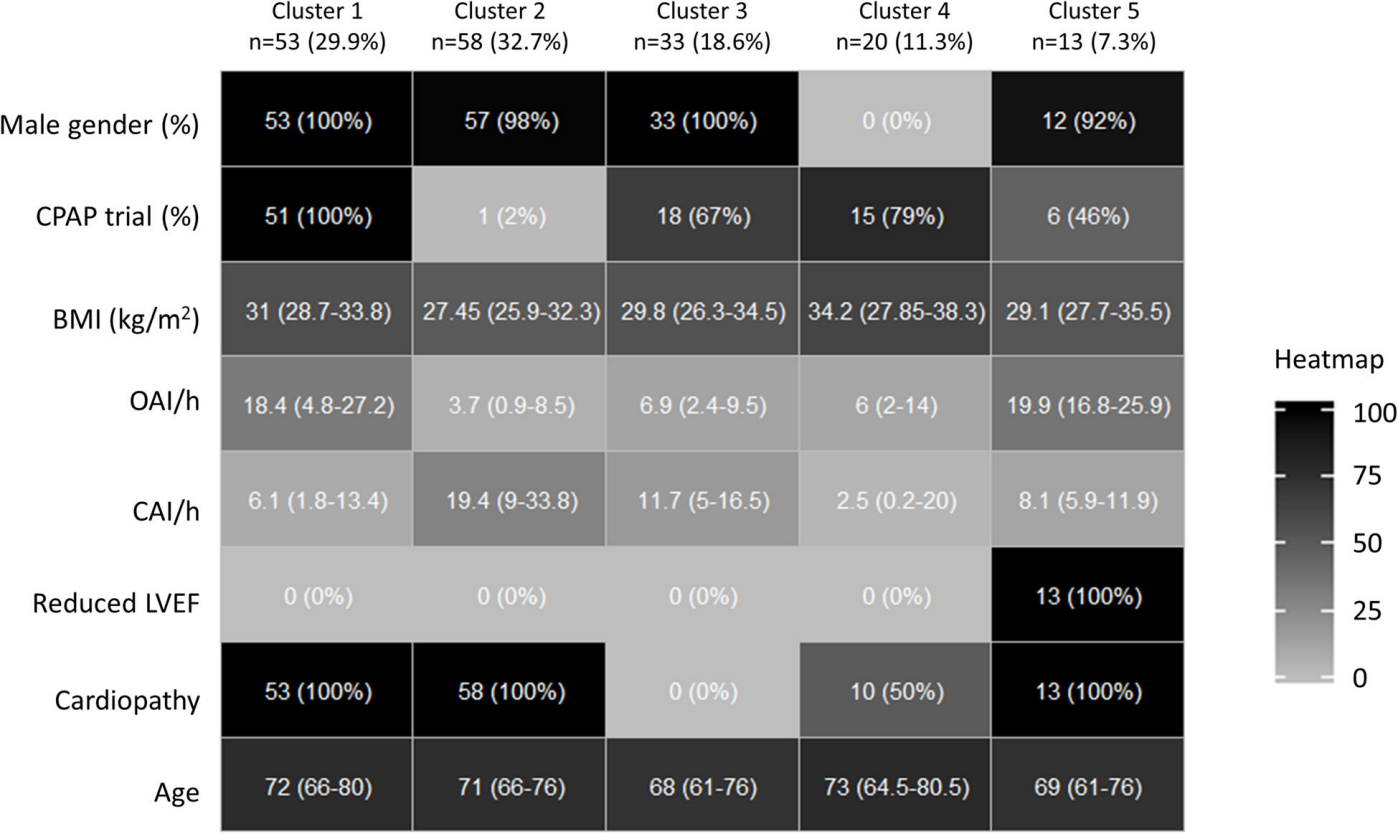
Heat map with descriptive statistics for variables used to construct *k*-means clusters. Quantitative variables were summarized using medians, [IQ25–75] and (min – max), while categories were described by numbers and (%). Each line represents a variable color-coded from 0 (white; minimum observed value) to 100% (black; maximum observed value). BMI: Body Mass Index; CAI: Central Apnea Index; CPAP: Continuous Positive Airway Pressure; LVEF: left ventricular ejection fraction; OAI: Obstructive Apnea Index

Five patient clusters were determined and Fig. 1 presents a heatmap indicating descriptive statistics for each “construction variable” per cluster. These variables were used to create the clusters, and so logically differed.

The first cluster is composed of male patients initialy treated with a CPAP device for a mixed sleep apnea syndrome (with a majority of obstructive apnea); these patients present a cardiopathy without reduced LVEF. Cluster 2 is predominantly composed of male patients without an initial CPAP trial for a mixed sleep apnea syndrome (with a majority of central apnea); these patients also present a cardiopathy without altered LVEF. Cluster 3 is composed of male patients without cardiopathy, but a mixed sleep apnea syndrome (with a majority of central apnea); 66.67% of these patients had an initial CPAP trial. Cluster 4 is composed of female patients with an initial CPAP trial (78.95%) for a mixed sleep apnea syndrome (with a majority of hypopnea); these patients present an increased BMI, and 50% a cardiopathy without altered LVEF. Cluster 5 is predominantly composed of male patients with reduced LVEF, initialy treated with a CPAP device (46.15%) for a mixed sleep apnea syndrome (with a majority of obstructive apnea).

Additional file 4 and Table 3 respectively summarize the general/sleep characteristics and the ASV-settings data for these patient clusters. Statistical differences between clusters were found for the following “non-constructive” variables: fixed EPAP, minimum IPS and auto backup RR. In particular, reduced-LVEF-cluster-5 was associated with a significantly lower fixed EPAP level in comparison with clusters 1 (*p* = 0.029) and 4 (*p* = 0.007). Auto-EPAP usage also differed between the 5 clusters (*p* < 0.001) with pairwise comparisons demonstrating higher auto-EPAP use in the “no cardiopathy”-cluster-3 in comparison with “presence of cardiopathy”-clusters 1 and 2 (respectively *p* = 0.006 and < 0.001). Versus cluster-3, cluster-1 is characterized by a higher OAI/h and a higher initial CPAP-trial rate (respectively *p* = 0.04 and < 0.001).

**Table 3:** Adaptive servo-ventilation settings for the OTRLASV population based on *k*-means clusters

	Total ***n*** = 177 (100%)	Cluster 1 ***n*** = 53 (29.9%)	Cluster 2 ***n*** = 58 (32.7%)	Cluster 3 ***n*** = 33 (18.6%)	Cluster 4 ***n*** = 20 (11.3%)	Cluster 5 ***n*** = 13 (7.3%)	***P***
**Fixed EPAP**^k^ (cmH_2_O)	*n* = 121	*n* = 39	*n* = 46	*n* = 12	*n* = 15	*n* = 9	
	6.00	8.00 ^e, i^	5.00 ^b, e, j,^	6.50 ^j^	8.00 ^b, d^	5.00 ^d, i^	< 0.001
	[5.00–9.00]	[5.00–10.00]	[4.00–6.00]	[5.00–9.00]	[6.00–12.00]	[5.00–6.00]	
	(4.00–14.00)	(4.00–14.00)	(4.00–13.00)	(4.00–12.00)	(4.00–14.00)	(4.00–7.00)	
**Auto-EPAP** (cmH_2_O)^k^	*n* = 56	*n* = 14	*n* = 12	*n* = 21	*n* = 5	*n* = 4	
**EPAPmin**	4.00	4.50	4.00	4.00	4.00	5.00	0.529
	[4.00–5.50]	[4.00–6.00]	[4.00–4.50]	[4.00–6.00]	[4.00–5.00]	[4.50–6.50]	
	(4.00–13.00)	(4.00–13.00)	(4.00–7.00)	(4.00–10.00)	(4.00–8.00)	(4.00–8.00)	
**EPAPmax**	10	10	10	10	9	7	0.211
	[8.00–12.00]	[8.00–12.00]	[8.00–10.00]	[9.00–12.00]	[8.00–10.00]	[6.00–9.50]	
	(3.00–18.00)	(3.00–15.00)	(5.00–10.00)	(5.00–18.00)	(8.00–12.00)	(5.00–12.00)	
**IPS** (cmH_2_O)^k^	*n* = 176	*n* = 53	*n* = 57	*n* = 33	*n* = 20	*n* = 13	
**IPSmin**	3.00	3.00 ^e^	3.00 ^e, j^	0.00 ^j^	3.00	3.00	< 0.001
	[0.00–3.00]	[3.00–3.00]	[3.00–6.50]	[0.00–3.00]	[0.00–3.00]	[3.00–4.00]	
	(0.00–15.00)	(0.00–7.5.00)	(0.00–15.00)	(0.00–8.00)	(0.00–10.00)	(0.00–10.00)	
**IPSmax**	10	10	10	10	10	10	0.063
	[9.00–10.00]	[10.00–10.00]	[10.00–12.00]	[10.00–11.00]	[8.00–10.00]	[8.00–10.00]	
	(3.00–20.00)	(5.00–15.00)	(3.00–20.00)	(5.00–15.00)	(5.00–15.00)	(5.00–13.00)	
**Pmax** (cmH_2_O)^k^	*n* = 129	*n* = 42	*n* = 37	*n* = 28	*n* = 15	*n* = 7	
	19	19	19	20.00	18.00	17.00	0.912
	[16.00–21.00]	[17.00–21.00]	[15.00–22.00]	[15.00–23.50]	[15.00–20.00]	[13.00–20.00]	
	(11.00–25.00)	(12.00–25.00)	(12.00–25.00)	(11.00–25.00)	(14.00–25.00)	(12.00–25.00)	
**Fixed backup RR**^k^ (cycle/min)	*n* = 39	*n* = 5	*n* = 25	*n* = 3	*n* = 2	*n* = 4	
	12	12	11.5	11.5	11.5	11.5	0.856
	[11.00–12.00]	[12.00–12.00]	[10.00–12.50]	[10.00–12.00]	[10.00–12.00]	[10.00–12.00]	
	(8.00–16.00)	(8.00–14.00)	(10.00–16.00)	(10.00–22.00)	(10.00–22.00)	(10.00–22.00)	
**Auto backup RR**^k^	*n* = 174	*n* = 52	*n* = 57	*n* = 33	*n* = 20	*n* = 12	
	135 (77.59%)	47(90.38%) ^e^	32 (56.14%) ^e, j^	30 (90.91%) ^j^	18 (90.00%)	8 (66.67%)	< 0.001
**Slope level**^l^	*n* = 42	*n* = 5	*n* = 26	*n* = 3	*n* = 3	*n* = 5	
	2	2	2	1	3	3	0.146
	[2.00–3.00]	[1.00–2.00]	[2.00–3.00]	[1.00–2.00]	[2.00–3.00]	[2.00–3.00]	
	(1.00–6.00)	(1.00–3.00)	(1.00–6.00)	(1.00–2.00)	(2.00–3.00)	(2.00–3.00)	
**Ti min**^l^ (second)	*n* = 38	*n* = 5	*n* = 24	*n* = 3	*n* = 2	*n* = 4	
	1.65	1.60	1.70	1.50	1.45	1.60	0.736
	[1.40–1.80]	[1.40–1.80]	[1.40–1.90]	[1.00–1.60]	[1.20–1.70]	[1.35–1.85]	
	(1.00–2.20)	(1.00–2.10)	(1.00–2.20)	(1.00–1.60)	(1.20–1.70)	(1.20–2.00)	
**Interface**	*n* = 177	*n* = 53	*n* = 58	*n* = 33	*n* = 20	*n* = 135	
Oronasal	89 (50.28%)	29 (54.72%)	27 (46.55%)	16 (48.48%)	12 (60.00%)	(38.46%)	0.687
Nasal	81 (45.76%)	23 (43.40%)	29 (50.00%)	15 (45.45%)	6 (30.00%)	8 (61.54%)	0.427
Nasal Pillows	9 (5.08%)	1 (1.89%)	4 (6.9%)	2 (6.06%)	2 (10%)	0 (0.00%)	0.492
**Heated humidifier**	*n* = 177	*n* = 53	*n* = 58	*n* = 33	*n* = 20	*n* = 13	
	127 (71.75)	43 (81.13)	34 (58.62)	26 (78.79)	15 (75.00)	9 (69.23)	0.092
**Heated breathing tube**	*n* = 177	*n* = 53	*n* = 58	*n* = 33	*n* = 20	*n* = 13	
	47 (26.55)	14 (26.42) ^f^	8 (13.79) ^j^	19 (57.58) ^f, j, h^	5 (25.00)	1 (7.69) ^h^	< 0.001

Quantitative variables were summarized using medians, [IQ_25_–_75_] and (min – max), while categories were described by numbers and (%). The total percentage for interfaces is > 100% because multiple interfaces were used by 2 patients

Significant (*p* < 0.05) post-hoc pairwise comparisons after Holm correction (within lines) were presented using labels ^a, b, c, d, e, f, g, h, I, j^. Label ^a^ indicates a significant difference between Cluster 1 and Cluster 4, label ^b^ indicates a significant difference between Cluster 2 and Cluster 4, label ^c^ indicates a significant difference between Cluster 3 and Cluster 4, label ^d^ indicates a significant difference between Cluster 4 and Cluster 5, label ^e^ indicates a significant difference between Cluster 1 and Cluster 2, label ^f^ indicates a significant difference between Cluster 1 and Cluster 3, label ^g^ indicates a significant difference between Cluster 2 and Cluster 5, label ^h^ indicates a significant difference between Cluster 3 and Cluster 5, label ^i^ indicates a significant difference between Cluster 1 and Cluster 5, label ^j^ indicates a significant difference between Cluster 2 and Cluster 3

*CSA* Central sleep apnea, *EPAP* Expiratory positive airway pressure, *IPS* Inspiratory pressure support, *max* maximum, *min* minimum, *OSA* Obstructive sleep apnea, *Pmax* maximum pressure, *RR* Respiratory rate, *TECSA* Treatment emergent central sleep apnea, *Ti min* inspiratory support minimum pressurization time

^k^For ASV-setting variables shared by both manufacturers, the data were pooled (EPAP (fixed or minimum/maximum); IPS (minimum and maximum), Pmax, RR or auto RR). ^l^For slope level and minimum inspiratory time, the data concern only Philips Respironics devices

**Fig. 2:**
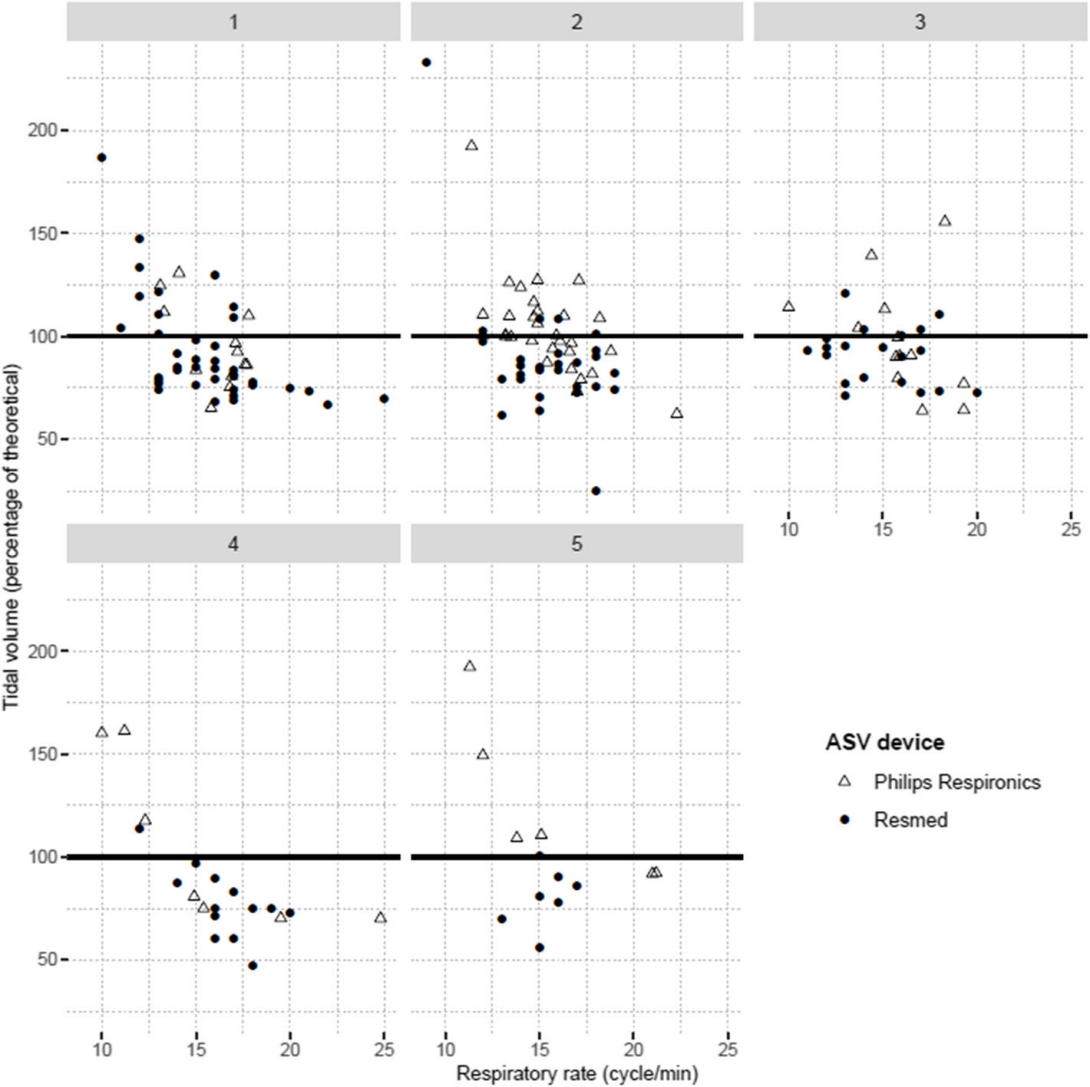
Individual tidal volume (% theoretical) versus respiratory rate measures for each cluster. For each patient, the theoretical tidal volume is calculated using an 8 ml/kg formula and the ideal weight is calculated according to the Lorentz equation. Measured tidal volume is obtained by dividing the measured minute-ventilation by respiratory rate. Note that for Philips Respironics® devices, the minute-ventilation is expressed as “mean” in the manufacturer software whereas for ResMed® devices, the minute-ventilation is expressed as “median” (thus preventing direct comparisons between these device types). Data are collected using the manufacturer’s software for a continuous 6-month period

Table 4 depicts MV depending on ASV-monitoring-based and cluster-based groups. For ASV-F (Philips Respironics® Devices) and ASV-V (Resdmed® devices), cluster MV differences exist (respectively *p* = 0.002 and *p* < 0.001), but the latter are not identical. Indeed, for ASV-F treated patients, reduced-LVEF-cluster-5 was associated with a higher MV than clusters 1 (*p* = 0.019) and 4 (*p* = 0.001), and cluster-2 was associated with higher MV than cluster-4 (*p* = 0.016). For ASV-V treated patients, a lower MV exists for cluster-4 versus clusters 1 (*p* < 0.001), 2 and 3 (both *p* = 0.004). Figure 2 depicts per-patient respiratory rates versus tidal volume (as a percentage of theorical tidal volume). All clusters demonstrate the presence of standardized tidal volumes over 100%, indicating a corresponding, homogenously-present risk for hyperventilation. Additional file 5 reports ASV-software measured data for EPAPs, IPSs and RR. Significant cluster differences again exist for Philips Respironics devices, but not for ResMed devices. In particular, the measured mean 90th EPAP is higher for cluster-3 versus cluster-2 (*p* = 0.001) and mean IPS is higher for cluster-2 versus cluster-1 (0.043). For a given manufacturer brand, there is no significant difference between clusters for AHI_flow_, leaks and ASV-observance.

**Table 4:** Minute-ventilation mean/medians for adaptive servo-ventilation treatment according to *k*-means clusters and device type

	Total ***n*** = 177 (100%)	Cluster 1 ***n*** = 53 (29.9%)	Cluster 2 ***n*** = 58 (32.7%)	Cluster 3 ***n*** = 33 (18.6%)	Cluster 4 ***n*** = 20 (11.3%)	Cluster 5 ***n*** = 13 (7.3%)	***P***
**Philips Respironics®**	*n* = 68	*n* = 12	*n* = 30	*n* = 13	*n* = 7	*n* = 6	0.002
Mean MV (l/min)*	7.95	7.85 ^i^	8.30 ^b^	7.90	6.20 ^b, d^	10.45 ^d, i^	
*ASV flow-monitored devices.*	[7.20–9.30]	[6.65–8.25]	[7.30–9.30]	[7.30–8.90]	[5.30–7.80]	[9.90–11.20]	
	(4.90–14.80)	(5.10–10.80)	(6.40–11.70)	(5.80–14.80)	(4.90–7.80)	(8.30–11.30)	
**ResMed®**	*n* = 107	*n* = 39	*n* = 28	*n* = 20	*n* = 13	*n* = 7	< 0.001
Median MV (l/min)*	6.90	7.30 ^a^	6.85 ^b^	7.00 ^c^	5.50 ^a, b, c^	6.60	
*ASV volume-monitored devices.*	[6.00–7.80]	[6.60–8.30]	[6.35–7.65]	[6.00–7.45]	[5.00–5.90]	[5.10–7.80]	
	(2.50–11.40)	(5.40–10.30)	(2.50–11.40)	(4.60–10.60)	(3.60–6.90)	(3.80–9.80)	

Variables were summarized using medians, [IQ_25_–_75_] and (min – max)

*Note that for Philips Respironics® devices, the minute-ventilation is expressed as “mean” in the manufacturer software whereas for ResMed® devices, the minute-ventilation is expressed as “median” (thus preventing direct comparisons between these device types)

*ASV* Adaptive servo-ventilation, *MV* minute-ventilation

Significant (*p* < 0.05) post-hoc pairwise comparisons after Holm correction (within lines) were presented using labels ^a, b, c, d, I^. Label ^a^ indicates a significant difference between Cluster 1 and Cluster 4, label ^b^ indicates a significant difference between Cluster 2 and Cluster 4, label ^c^ indicates a significant difference between Cluster 3 and Cluster 4, label ^d^ indicates a significant difference between Cluster 4 and Cluster 5, label ^i^ indicates a significant difference between Cluster 1 and Cluster 5

## Discussion

The SERVE-HF study raised serious concerns about ASV safety [7]. It was hypothesized that the hyperventilation associated with high-pressure ASV default settings in SERVE-HF could explain the higher cardiovascular mortality observed [8–10]. To the best of our knowledge, we report herein in long-term patients treated with different ASV servo-controlled monitoring type, the first real life description of differences in ASV-settings and the resulting minute-ventilations/therapeutic pressures associated with unsupervised patient-clusters. Our multicentre study spotlights four main results: i) certain clusters are associated with different ASV-settings; ii) certain clusters are associated with differences in MV; iii) MV differences between ASV-F and ASV-V are not homogenously distributed across clusters, suggesting specific cluster and ASV-algorithm interactions; iv) individual data suggest that at the patient-level, the risk of hyperventilation is present regardless of cluster or ASV servo-controlled monitoring type.

### ASV-setting associations with initial SDB-diagnostic-based groups and aetiology/comorbidity-based clusters

To date, there are no ASV-settings mentioned in relevant recommendations [29, 30]. In pratice, for ventilation-naive patients, clinicians have the choice between manufacturer default ASV-settings and patient-individualized ASV-settings. There are also no contemporary long-term clinical trials comparing these two modalities, but results from a bench test study are in favor of manually implementing individualized ASV-settings [2]. For previously CPAP-treated patients, starting the ASV-setting titration at or near the CPAP level was proposed (the EPAP pressure level was adjusted up to a maximum of 10 cmH_2_O and the manufacturer default inspiratory pressure range was allowed to vary between 5 and 10 cmH_2_O above the EPAP) [32].

In our study, the 8 expert clinicians were free to adjust ASV-settings as they saw fit. We report herein that patient ASV-settings do not differ between initial SDB-diagnostic-based groups (i.e. OSA, CSA or TECSA). This is not surprising if we consider that: i) our ASV-population consists of long-term treated-patients (median ASV duration is 2.88 years, 25% treated for more than 4.96 years); ii) regardless of the the ASV-initiation date, we have previously reported that 34.4% of the patients were monitored (polygraphy/oximetry) for 6 months preceding inclusion with a consecutive ASV-settings change performed among 18.2% of them [24]; and iii) the initial difference between SDB-diagnostic-based groups was the initial apnea pattern, whereas these patients also presented hypopnea events with undefined central or obstructive patterns.

Our study spotlights the real-life choices made by expert-clinicians. First, for patients presenting both reduced-LVEF and an initial obstructive apnea diagnosis pattern (Cluster-5 in our study), the majority of our experts chose “safety first,” i.e. they chose a significantly lower EPAP level in comparison with clusters paradoxically characterized by a lower OAI/h. The latter was likely meant to prevent deleterious hemodynamic effects on cardiac output [16, 17, 33, 34]. Importantly for cluster-5, the choice of a lower EPAP was not associated with a significant increase in the residual IAH_flow_, suggesting that a “safety-first” attitude did not sacrifice efficacity for these patients. The second interesting choice concerns the auto-EPAP usage in real life by the experts. In the latter mode, the EPAP is automatically adjusted by specific manufacturer algorithms meant to correct obstructive disordered breathing [3, 35]. To date, in terms of correcting obstructive events, the superiority of the auto-EPAP mode over the fixed-EPAP mode for ASV-device has not been demonstrated [2, 21, 36, 37]. In this context, it is important to underline that the auto-EPAP-usage by the experts was significantly different between the five clusters, with pairwise comparisons demonstrating a higher auto-EPAP use in the “no cardiopathy”-cluster-3 in comparison with “presence of cardiopathy”- clusters 1 and 2. Again, this suggests a “safety-first” attitude among the expert clinicians participating in the study and is supported by AASM and French guidelines recommending not using auto-Positive-Airway-Pressure devices in CHF populations [38–40]. The OTRLASV population is characterized by 59.36% CHF and 30.46% atrial fibrillation. For these cardiologic phenotypes and ASV-treated patients, the deployment of auto-EPAP requires a higher level of evidence. In this context, the scientific community is eagerly awaiting the results of the ADVENT-HF trial [41].

Pending the results of future ASV-studies, our data suggest that “safety first” strategies are currently driving physician-chosen ASV-settings (i.e. a low EPAP level and a low auto-EPAP usage for patients with a reduced LVEF and cardiopathy, respectively). Pragmatically, it would be helpful i) to verify the impact of ASV-settings on heart function using echocardiography [17, 19, 20], digital photoplethysmography [42] or bioimpedance-based monitorings [19]; ii) to perform night-monitoring of ASV-settings with polysomnography, transcutaneous capnometry and simultaneous non-invasive measures of diastolic blood pressure / heart rate variation in order to evaluate not only ASV effects on AHI and sleep but also the absence of negative ASV effects on sympathovagal balance [22].

### Relationships between minute-volume, SDB-diagnosis-based groups, aetiology/cormorbidity clusters, and flow- versus volume-controlled monitoring

In their short-term study (mean follow-up time was 8.2 ± 3.0 weeks), Westhoff and Litterst describe the relationship between patient phenotypes and the associated MV resulting from an ASV-V device (Auto-CS-2 ResMed®) [28]. Neither MV nor therapeutic pressure differences were observed between TECSA (without elevated BNP/NT-pro-BNP) versus mixed apnea patients (with predominantly central pattern and elevated BNP/NT-pro-BNP). Based on our initial SDB-diagnostic-based group comparisons, we report similar results for both ASV-V and ASV-F devices.

Knitter et al. previously attempted to describe MV differences between ASV-F and ASV-V devices in TECSA patients with preserved LVEF [4]. The latter study consisted of a randomized cross-over study comparing 4 devices (each used for one night only) and concluded that the ResMed S7 VPAP Adapt (an ASV-V device) was associated with a higher MV than ASV-F devices. The authors directly compared ASV-F and ASV-V devices, which is a major difference with the present study as we considered it statistically impossible to compare the MV measures provided by the manufacturer software (because one is expressed as a mean (Philips Respironics® devices, ASV-F) and the other as a median (ResMed® device, ASV-V)). A further statistical limitation is that these results are expressed as “measured MV or measured tidal volume”, whereas theoretical percentages would be more appropriate. Indeed, at the individual patient level, hyperventilation status is better determined by a theoretical percentage of what is normal for a patient, and not by brute measures. This is why our study additionally presents theoretical tidal volumes based on ideal weight and an expected 8 ml/kg theoretical tidal volume. To date, it is difficult to go further in the interpretation of our results because there is no robust evidence base defining the patient normal MV. It is however quite surprising to observe that a servo-controlled volume monitoring device can be associated with hyperventilation whereas its algorithm targets only 90 to 95% of the recent average ventilation calculated [3]. For some patients, one must consider the possibility that a raise in MV could be indicative of a CHF exacerbation, independent of the device. In this regard, paying attention not only to MV but also to the respiratory rate and the percentage of respiratory cycles triggered by the patient may have an interest similar to that described for patients with exacerbating severe chronic obstructive pulmonary disease [43].

Although we did not perform direct comparisons between ASV-F and a ASV-V devices, our K-means clustering analysis demonstrated that MV differences between ASV-F and ASV-V are not homogenously distributed across clusters, indicating a cluster and ASV-algorithm interaction. The latter observation is important when considering the ASV debate over “class-effects” versus “device-effects” as an explanation for the cardiovascular mortality seen in the SERVE-HF study [8–10]. Our results are in favour of the simultaneous presence of both types of effect. The occurrence of a higher-than-expected tidal volume on an individual basis, regardless of cluster or the type of ASV algorithm used, emphasizes the crucial need for individual MV monitoring. In this regard, MV telemonitoring as part of a safety-first strategy deserves consideration, as least for patients with reduced-LVEF (like cluster-5 in the present study, which has higher MV when patients are treated with ASV-F).

### Limits of the study

Because real-life data describing ASV-settings and their associated flow- versus volume- controlled monitoring are sparse, our study was exploratory and descriptive in nature, without predefined hypotheses to be tested. Assessing the effects or interactions between ASV-settings, servo-controlled-flow or -volume monitoring types, and patient-phenotypes is complicated considering that the patient characteristics and the physician prescriptions are scalable. However, our study design (clinically stable and long-term treated patients, annual planned consultation corresponding to the inclusion date) limits in as much as possible such confounders.

Our patients were included from March 13, 2015 to December 31, 2016. The SERVE-HF safety announcement was made on May 13, 2015. As a consequence, the ASV-data collected result from a mix of before and after SERVE-HF physician behaviors (with a majority of post-SERVE-HF inclusions). In addition, we were unable to generate a complete patient-group with a SERVE-HF like pattern because of the post SERVE-HF restrictions by regulators that occurred only 3 months after our study started. The absence of a SERVE-HF-group limits a direct comparison between the latter and our three groups based on initial diagnosed sleep-disordered breathing. For similar reasons, it was impossible for us to constitute a group of patients with a SERVE-HF pattern and undergoing CPAP treatment. Indeed, the indication of a CPAP treatment for these patients became consensual in France in 2017 subsequent to the European Respiratory Task Force Report [30].

In relation with our cross-sectional study design/inclusion criteria, another study limitation is that we are able to report only the hyperventilation risk in real life ASV-treated patients, and not longitudinal cardiovascular mortality. In addition, the reader should keep in mind that this cross-sectional study is a non-randomized real life study with potential unknown sources of bias. To limit this risk, a multicentre design without predefined ASV-rules or ASV-brand requirements was used. An important further limitation results from the impossibility of making direct comparisons between ASV-F and ASV-V. Because of our long-term design, it was impossible for us to deploy an independent pneumotachograph MV and therapeutic pressure measures, and we therefore used manufacturer software as in previous clinical publications [4, 28]. However, based on the ASV bench-study published by Zhu et al. [2], which used an independent pneumotachograph and demonstrated device-differences in MV and therapeutic pressures, one can assume that such machine related heterogeneity exists in real life.

## Conclusion

Real-life, long-term ASV-settings are not associated with the initial SDB-diagnosis-based group but are rather associated with combinations of baseline and follow-up variables wherein reduced-LVEF remains clinically meaningful. EPAP-pressures and auto-EPAP usage are lower in the reduced-LVEF cluster even when a predominant obstructive pattern is present. Crucially, the latter choices did not result in increased residual IAH_flow_. MV differences exist between clusters. Simultaneously, MV differences between devices (ASV-F versus ASV-V) vary with clusters. Importantly, a higher-than-expected tidal volume can occur independently of ASV monitoring type or cluster, suggesting a need for individual MV telemonitoring in ASV patients.

## Supplementary information

**Additional file 1.** Study flow chart. ASV-settings/software measured data were analysed i) between initial sleep-disordered-breathing diagnostic based groups (CSA, OSA, and TECSA) and ii) between unsupervised based groups (created via a clustering algorithm). AHC: Ascending Hierarchical Classification; ASV: Adaptive Servo-Ventilation; CSA: Central Sleep Apnea; OSA: Obstructive Sleep Apnea; OTRLASV: Observational Transversal Real-life Study of ASV; SDB: Sleep Disordered Breathing; TECSA: Treatment Emergent Central Sleep Apnea.

**Additional file 2.** Initial sleep-disordered-breathing diagnostic based groups For the SDB group analysis, three patient groups were generated (the central sleep apnea (CSA), obstructive sleep apnea (OSA) and treatment-emergent central sleep apnea (TECSA) groups). In line with our recent publication and those from Malfertheiner et al. [1, 2], we chose to differentiate central versus obstructive groups using the predominant apnea pattern during the initial polygraphy (PG) or polysomnography (PSG) diagnosis. Central apnea was scored if respiratory effort was absent. This latter criteria was chosen because it represented a consensus between the different centers and recommendations for scoring. Patients with an initial diagnosis of OSA treated with Continuous Positive Airway Pressure (CPAP) but secondarily treated with ASV were classified in the (TECSA) group. The detailed algorithm is included in our initial publication [1].

**Additional file 3.** Software-measured data for the 6 months preceding the study inclusion. Philips Respironics® and ResMed® (grey line) device-reported outcomes based on initial sleep-disordered-breathing diagnostic groups.

**Additional file 4.** General and sleep characteristics of the OTRLASV population and for k-means clusters.

**Additional file 5.** Software measured data for the last 6 months preceding the study inclusion. Philips Respironics® and ResMed® (grey line) device-reported outcomes based on the cluster analysis.

## Data Availability

The datasets used and/or analyzed during the current study are available from the corresponding author on reasonable request.

## Abbreviations

AASM: American Academy of Sleep Medicine
AHI: Apnea Hypopnea Index
AHI_flow_: Residual Apnea–Hypopnea-Index measured by the ASV device
ASV: Adaptive Servo-Ventilation
ASV-F: Flow monitored ASV
ASV-V: Volume monitored ASV
BMI: Body Mass Index
CAI: Central Apnea Index
CHF: Chronic Heart Failure
CPAP: Continuous Positive Airway Pressure
CSA: Central Sleep Apnea
ESS: Epworth Sleepiness Scale
HCA: hierarchical clustering approach
HI: Hypopnea Index
IQ_25–75_: Medians and interquartile ranges
LVEF: Left Ventricular Ejection Fraction
MAI: Mixed Apnea Index
OAI: Obstructive Apnea Index
OSA: Obstructive Sleep Apnea
OTRLASV: Observational Transversal Real-life Study of ASV
PG: Respiratory polygraphy
PSG: Polysomnography
TECSA: Treatment Emergent Central Sleep Apnea
SA: Sleep Apnea
SDB: Sleep-Disordered Breathing
SD: Standard deviations
WP: Whole population
