# Supplementary material for: Patterns of adaptive servo-ventilation settings in a real-life multicenter study: pay attention to volume! Adaptive servo-ventilation settings in real-life conditions
Source: Respir Res. 2020 Sep 21;21:243. doi: 10.1186/s12931-020-01509-7 (PMC7507637; doi:10.1186/s12931-020-01509-7)

**Additionnal file 1:** Study flow chart

ASV-settings/software measured data were analysed i) between initial sleep-disordered-breathing diagnostic based groups (CSA, OSA, and TECSA) and ii) between unsupervised based groups (created via a clustering algorithm).

AHC: Ascending Hierarchical Classification; ASV: Adaptive Servo-Ventilation; CSA: Central Sleep Apnea; OSA: Obstructive Sleep Apnea; OTRLASV: Observational Transversal Real-life Study of ASV; SDB: Sleep Disordered Breathing; TECSA: Treatment Emergent Central Sleep Apnea.


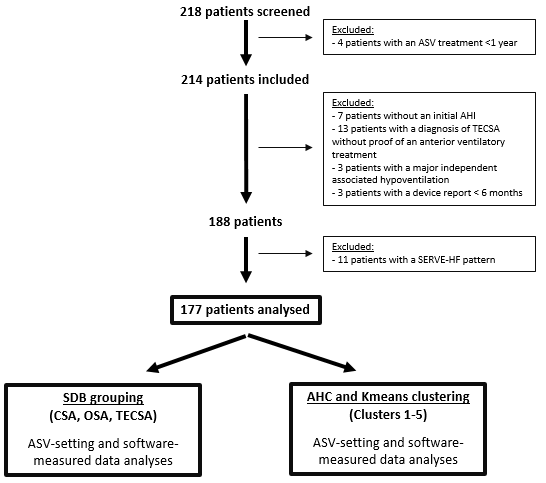

Supplement: Supplementary file 1 — Additional file 1. Study flow chart. ASV-settings/software measured data were analysed i) between initial sleep-disordered-breathing diagnostic based groups (CSA, OSA, and TECSA) and ii) between unsupervised based groups (created via a clustering algorithm). AHC: Ascending Hierarchical Classification; ASV: Adaptive Servo-Ventilation; CSA: Central Sleep Apnea; OSA: Obstructive Sleep Apnea; OTRLASV: Observational Transversal Real-life Study of ASV; SDB: Sleep Disordered Breathing; TECSA: Treatment Emergent Central Sleep Apnea. [file 12931_2020_1509_MOESM1_ESM.docx]
