# Supplementary material for: Patterns of adaptive servo-ventilation settings in a real-life multicenter study: pay attention to volume! Adaptive servo-ventilation settings in real-life conditions
Source: Respir Res. 2020 Sep 21;21:243. doi: 10.1186/s12931-020-01509-7 (PMC7507637; doi:10.1186/s12931-020-01509-7)
Supplement: Supplementary file 2 — Additional file 2. Initial sleep-disordered-breathing diagnostic based groups For the SDB group analysis, three patient groups were generated (the central sleep apnea (CSA), obstructive sleep apnea (OSA) and treatment-emergent central sleep apnea (TECSA) groups). In line with our recent publication and those from Malfertheiner et al. [1, 2], we chose to differentiate central versus obstructive groups using the predominant apnea pattern during the initial polygraphy (PG) or polysomnography (PSG) diagnosis. Central apnea was scored if respiratory effort was absent. This latter criteria was chosen because it represented a consensus between the different centers and recommendations for scoring. Patients with an initial diagnosis of OSA treated with Continuous Positive Airway Pressure (CPAP) but secondarily treated with ASV were classified in the (TECSA) group. The detailed algorithm is included in our initial publication [1]. [file 12931_2020_1509_MOESM2_ESM.docx]

**Additional file 2:** Initial sleep-disordered-breathing diagnostic based groups

For the SDB group analysis, three patient groups were generated (the central sleep apnea (CSA), obstructive sleep apnea (OSA) and treatment-emergent central sleep apnea (TECSA) groups). In line with our recent publication and those from Malfertheiner et al. [1,2], we chose to differentiate central versus obstructive groups using the predominant apnea pattern during the initial polygraphy (PG) or polysomnography (PSG) diagnosis. Central apnea was scored if respiratory effort was absent. This latter criteria was chosen because it represented a consensus between the different centers and recommendations for scoring. Patients with an initial diagnosis of OSA treated with Continuous Positive Airway Pressure (CPAP) but secondarily treated with ASV were classified in the (TECSA) group. The detailed algorithm is included in our initial publication [1].

**Références**

1. Jaffuel D, Philippe C, Rabec C, Mallet J-P, Georges M, Redolfi S, et al. What is the remaining status of adaptive servo-ventilation? The results of a real-life multicenter study (OTRLASV-study) : Adaptive servo-ventilation in real-life conditions. Respir Res. 2019;20:235.

2. Malfertheiner MV, Lerzer C, Kolb L, Heider K, Zeman F, Gfüllner F, et al. Whom are we treating with adaptive servo-ventilation? A clinical post hoc analysis. Clin Res Cardiol. 2017;106:702–10.
