# Supplementary material for: Patterns of adaptive servo-ventilation settings in a real-life multicenter study: pay attention to volume! Adaptive servo-ventilation settings in real-life conditions
Source: Respir Res. 2020 Sep 21;21:243. doi: 10.1186/s12931-020-01509-7 (PMC7507637; doi:10.1186/s12931-020-01509-7)
Supplement: Supplementary file 3 — Additional file 3. Software-measured data for the 6 months preceding the study inclusion. Philips Respironics® and ResMed® (grey line) device-reported outcomes based on initial sleep-disordered-breathing diagnostic groups. [file 12931_2020_1509_MOESM3_ESM.docx]

**Additional file 3.**

Software-measured data for the 6 months preceding the study inclusion. Philips Respironics® and ResMed® (grey line) device-reported outcomes based on initial sleep-disordered-breathing diagnostic groups.

|  | **Total**  **n=177 (100%)** | | **CSA group,**  **n=105 (59.3%)** | **OSA group,**  **n=36 (20.3%)** | **TECSA group,**  **n=36 (20.3%)** | **P** |
| --- | --- | --- | --- | --- | --- | --- |
| **Philips Respironics®** | 68 | | 36 (34.3%) | 18 (50.0%) | 14 (38.9%) | 0.246 |
| **ResMed®** | 109 | | 69 (65.7%) | 18 (50.0%) | 22 (61.1%) |  |
| **Mean EPAP** (cmH_2_O) | n=67  5.00  [4.30 – 6.00]  (4.00 – 11.90) | | n=35  4.80  [4.00 – 5.40]  (4.00 –9.60) | n=18  4.95  [4.60 – 6.60]  (4.00 – 11.00) | n=14  5.40  [5.00 – 7.90]  (4.00 – 11.90) | 0.124 |
| **Median EPAP** (cmH_2_O) | n=89  7.20  [6.00 – 10.00]  (4.00 – 14.00) | | n=51  8.00  [5.00 – 10.00]  (4.00 –14.00) | n=18  6.50  [6.00 – 8.00]  (5.00 – 12.00) | n=20  7.10  [6.30 – 9.00]  (5.00 – 14.00) | 0.552 |
| **Mean 90^th^ EPAP** (cmH_2_O) | n=67  5.20  [4.50 – 7.60]  (4.00 – 14.30) | | n=36  5.00  [4.25 – 6.20]  (4.00 – 11.90) | n=17  5.80  [5.00 – 7.60]  (4.00 – 11.00) | n=14  6.60  [5.00– 8.70]  (4.00 – 14.30) | 0.340 |
| **Median 95^th^ EPAP** (cmH_2_O) | n=90  7.95  [6.00 – 10.00]  (4.00 – 14.80) | | n=53  8.00  [6.20 – 10.00]  (4.00 – 14.80) | n=18  7.35  [6.00 – 9.50]  (5.00 – 12.00) | n=19  7.00  [6.00– 9.50]  (4.00 – 14.00) | 0.320 |
| **Mean IPS**  (cmH_2_O) | n=68  5.35  [1.90 – 8.20]  (0.50 – 16.40) | | n=36  6.20  [2.15 – 8.15]  (0.50 – 16.40) | n=18  4.80  [2.00 – 9.60]  (0.60 – 16.40) | n=14  3.25  [1.70 – 6.60]  (1.00– 9.20) | 0.339 |
| **Median IPS**  (cmH_2_O) | n=90  4.00  [3.50 – 4.60]  (1.10 – 14.10) | | n=52  4.20  [3.65 – 4.80]  (1.10 – 14.10) | n=18  3.55  [2.80 – 4.90]  (1.30 – 9.80) | n=20  3.95  [3.25– 4.40]  (1.10– 5.30) | 0.105 |
| **Mean 90^th^ IPS**  (cmH_2_O) | n=62  7.10  [4.40 – 8.90]  (1.00 – 14.90) | | n=31  7.60  [5.00 – 8.90]  (1.00 – 14.90) | n=17  6.00  [4.80 – 11.00]  (1.50 – 14.40) | n=14  6.75  [4.10 – 8.40]  (2.20 – 10.80) | 0.753 |
| **Median 95^th^ IPS**  (cmH_2_O) | n=90  7.10  [6.10 – 8.10]  (0.50 – 13.40) | | n=52  7.20  [6.30 – 8.45]  (0.50 – 13.40) | n=18  7.10  [4.90 – 7.70]  (2.40 – 12.40) | n=20  6.85  [5.65 – 7.75]  (2.00 – 9.00) | 0.317 |
| **Mean RR**  (cycle/min) | n=68  15.70  [13.75 – 17.10]  (10.00 – 24.80) | | n=36  15.80  [14.80 – 17.10]  (12.00 – 22.30) | n=18  13.90  [12.00 – 16.80]  (10.00 – 24.80) | n=14  15.75  [14.10 – 17.70]  (11.20 –21.00) | 0.378 |
| **Median RR**  (cycle/min) | n=108  15.50  [13.50 – 17.00]  (9.00 – 25.00) | | n=69  16.00  [14.00 – 17.00]  (9.00 – 25.00) | n=18  15.00  [14.00 – 17.00]  (11.00 – 20.00) | n=21  14.00  [13.00 – 16.00]  (12.00 –21.00) | 0.099 |
| **Mean**  **final AHI_flow_**  (n/h) | n=68  3.00  [2.00 – 5.45]  (0.20 – 22.60) | | n=36  2.65  [1.60 – 4.65]  (0.20 – 17.30) | n=18  3.30  [2.00 – 10.80]  (0.70– 22.60) | n=14  4.25  [3.30– 6.50]  (1.00 –15.40) | 0.046 |
| **Median final AHI_flow_**  (n/h) | n=109  1.10  [0.30 – 2.80]  (0.00 – 36.50) | | n=69  1.30  [0.30– 2.90]  (0.00 – 36.50) | n=18  0.90  [0.50 –1.90]  (0.00 – 17.20) | n=22  0.80  [0.20 – 2.80]  (0.00 –7.90) | *0.681* |
| **Mean important leaks (%)** | n=66  1.05 [0.3 – 3.8]  (0 – 66.) | | n=35  1 [0.1 – 3]  (0 – 30) | n=17  1 [0 .5 – 2.5]  (0 – 32.3) | n=14  2.2 [0.7 – 5.7]  (0 – 66) | 0.267 |
| **Median unintentional**  **leaks (l/min)** | n = 107  0 [0 – 6]  (0 – 93.6) | | n = 68  0 [0 – 6]  (0 – 93.6) | n 18  3 [0 – 16.8]  (0 – 56.4) | n = 21  0 [0 – 3.6]  (0 – 64.8) | 0.213 |
| **Mean**  **observance**  **(h/day)** | n=68  6.41  [5.15 – 7.40]  (2.68 – 11.65) | | n=36  6.31  [5.17 – 7.63]  (4.15 – 11.65) | n=18  6.37  [5.27 – 7.90]  (3.12 – 9.30) | n=14  6.52  [4.82 – 6.88]  (2.68 –8.12) | 0.608 |
| **Median observance**  **(h/day)** | n=109  6.55  [5.13 – 7.75]  (0.00 – 13.40) | | n=69  6.60  [5.85– 7.85]  (1.00 – 13.40) | n=18  6.38  [4.40 – 7.53]  (1.32 – 11.00) | n=22  6.37  [2.63 – 7.52]  (0.00 –8.47) | 0.181 |
| **Interface** | | n=177 | n=53 | n=58 | n=33 |  |

Variables were summarized using medians and [IQ_25_-_75_] and (min – max).

EPAP: expiratory positive airway pressure; IPS: inspiratory Pressure Support; max: maximum; min: minimum; RR: respiratory rate.
