# Supplementary material for: Patterns of adaptive servo-ventilation settings in a real-life multicenter study: pay attention to volume! Adaptive servo-ventilation settings in real-life conditions
Source: Respir Res. 2020 Sep 21;21:243. doi: 10.1186/s12931-020-01509-7 (PMC7507637; doi:10.1186/s12931-020-01509-7)
Supplement: Supplementary file 4 — Additional file 4. General and sleep characteristics of the OTRLASV population and for k-means clusters. [file 12931_2020_1509_MOESM4_ESM.docx]

**Additional file 4.** General and sleep characteristics of the OTRLASV population and for k-means clusters.

|  | **Total**  **n=177 (100%)** | **Cluster 1**  **n=53 (29.9%)** | **Cluster 2**  **n=58 (32.7%)** | **Cluster 3**  **n=33 (18.6%)** | **Cluster 4**  **n=20 (11.3%)** | **Cluster 5**  **n=13 (7.3%)** | **P** |
| --- | --- | --- | --- | --- | --- | --- | --- |
| Age (years) | 71  [65 - 77] | 72  [66.00 - 80.00] | 71  [66.00 - 76.00] | 68  [61.00 – 76.00] | 73  [64.50 - 80.50] | 69  [61.00 –76.00] | 0.171 |
| Gender |  |  |  |  |  |  | < 0.001 |
| Male | 155 (87.57%) | 53 (100%) ^a^ | 57 (98.28%) ^b^ | 33 (100%) ^c^ | 0 (0.00%)^a, b, c, d^ | 12 (92.31%) ^d^ |  |
| Female | 22 (12.43%) | 0 (0.00%) | 1 (1.72%) | 0 (0.00%) | 20 (100%) | 1 (7.69%) |  |
| BMI (kg/m^2^) | 29.90  [26.60-34.00] | 31 ^e^  [28.70 - 33.80] | 27.45 ^e^  [25.90 – 32.30] | 29.80  [26.30 – 34.50] | 34.20  [27.85 – 38.30] | 29.10  [27.70 –35.50] | 0.009 |
| **Initial sleep data** | | | | | | | |
| Initial AHI (n/h) | 50.00  [38.30 - 62.30] | 54.00  [40.00-76.00] | 46.00  [36.00-55.00] | 45.00  [36.00-56.60] | 54.50  [35.50-69.70] | 52.00  [42.00-54.60] | 0.092 |
| Initial OAI (n/h) | 7.70  [2.00 - 18.30] | 18.40 ^e, f^  [4.80-27.20] | 3.70 ^e, g^  [0.90-8.50] | 6.90 ^f, h^  [2.40-9.50] | 6.00 ^d^  [2.00-14.00] | 19.90 ^d, g, h^  [16.80-25.90] | <.001 |
| Initial CAI (n/h) | 10.75  [3.60 - 23.60] | 6.10 ^e^  [1.80-13.40] | 19.40 ^e, b, g^  [9.00-33.80] | 11.70  [5.00-16.50] | 2.50 ^b^  [0.2-20] | 8.10 ^g^  [5.90-11.90] | <.001 |
| Initial MAI (n/h) | 1.70  [0.00 - 5.00] | 2.70  [0.30-9.70] | 1.40  [0.00-4.90] | 1.90  [0.30-5.00] | 0.10  [0.00-1.70] | 0.00  [0.00-4.40] | 0.104 |
| Initial HI (n/h) | 16.00  [8.70 - 24.90] | 19.50 ^e^  [11.80-29.00] | 12.00 ^b, e^  [7.30-19.00] | 15.20  [10.50-25.05] | 25.20 ^b, d^  [12.70-44.00] | 13 ^d^  [8.00-17.00] | 0.003 |
| Initial ESS | 10.00  [6.00 - 13.50] | 10.00  [6.00-13.00] | 9.00  [4.00-12.00] | 11.00  [6.00-15.00] | 12.50  [2.00-14.00] | 12.00  [4.00-15.00] | 0.457 |
| CPAP trial before ASV initiation | 91 (54.82%) | 51 (100%) ^a, e, f, i^ | 1 (1.79%) ^b, e, g, j^ | 18 (66.67%) ^f, j^ | 15 (78.95%)^a, b^ | 6 (46.15%) ^g, i^ | <0.001 |
| Final ESS score | 6.00  [3.0-9.0] | 6.00  [3.0-10.50] | 5.00  [3.00-8.00] | 8.00  [4.00-10.00] | 4.00  [4.00-7.00] | 5.00  [2.00-7.00] | 0.273 |
| Cardiopathy | 134 (75.71) | 53 (100) ^a, f^ | 58 (100%) ^b, j^ | 0 (0.00%) ^c, f, h, j^ | 10 (50%) ^a, b, c, d^ | 13 (100%) ^d, h^ | <0.001 |
| Reduced LVEF | 13 (7.34%) | 0 (0.00%) ^i^ | 0 (0.00%) ^g^ | 0 (0.00%) ^h^ | 0 (0.00%) ^d^ | 13 (100%) ^d, g, h, i^ | <0.001 |

Quantitative variables were summarized using medians, [IQ25-75] and (min – max), while categories were described by numbers and (%).

Significant (p<0.05) post-hoc pairwise comparisons after Holm correction (within lines) were presented using labels ^a, b, c, d, e, f, g, h, I, j^. Label ^a^ indicates a significant difference between Cluster 1 and Cluster 4, label ^b^ indicates a significant difference between Cluster 2 and Cluster 4, label ^c^ indicates a significant difference between Cluster 3 and Cluster 4, label ^d^ indicates a significant difference between Cluster 4 and Cluster 5, label ^e^ indicates a significant difference between Cluster 1 and Cluster 2, label ^f^ indicates a significant difference between Cluster 1 and Cluster 3, label ^g^ indicates a significant difference between Cluster 2 and Cluster 5, label ^h^ indicates a significant difference between Cluster 3 and Cluster 5, label ^i^ indicates a significant difference between Cluster 1 and Cluster 5, label ^j^ indicates a significant difference between Cluster 2 and Cluster 3.

AHI: Apnea Hypopnea Index; BMI: Body Mass Index; CAI: Central Apnea Index; CPAP: Continuous Positive Airway Pressure; ESS: Epworth sleepiness scale; HI: Hypopnea Index, LVEF: left ventricular ejection fraction; MAI: Mixed Apnea Index; OAI: Obstructive Apnea Index.
