# Supplementary material for: Patterns of adaptive servo-ventilation settings in a real-life multicenter study: pay attention to volume! Adaptive servo-ventilation settings in real-life conditions
Source: Respir Res. 2020 Sep 21;21:243. doi: 10.1186/s12931-020-01509-7 (PMC7507637; doi:10.1186/s12931-020-01509-7)
Supplement: Supplementary file 5 — Additional file 5. Software measured data for the last 6 months preceding the study inclusion. Philips Respironics® and ResMed® (grey line) device-reported outcomes based on the cluster analysis. [file 12931_2020_1509_MOESM5_ESM.docx]

**Additional file 5.** Software measured data for the last 6 months preceding the study inclusion. Philips Respironics® and ResMed® (grey line) device-reported outcomes based on the cluster analysis**.**

|  | **Whole population,**  **n=177** | | | **Cluster 1**  **n=53 (30%)** | | | | **Cluster 2,**  **n=58 (33%)** | | | **Cluster 3,**  **n=33 (18.6%)** | | | **Cluster 4,**  **n=20 (11%)** | | | **Cluster 5**  **n=13 (7%)** | | | **P** | | |
| --- | --- | --- | --- | --- | --- | --- | --- | --- | --- | --- | --- | --- | --- | --- | --- | --- | --- | --- | --- | --- | --- | --- |
| **Philips Respironics®** | 68 (38.4%) | | | 12 (22.6%) ^e^ | | | | 30 (51.7%)^e^ | | | 13 (39.4%) | | | 7 (35%) | | | 6 (46.15%) | | | 0.031 | | |
| **ResMed®** | 109 (61.6%) | | | 41 (77.4%) ^e^ | | | | 28 (48.3%)^e^ | | | 20 (60.6%) | | | 13 (65%) | | | 7 (58.85%) | | |  | | |
| **Mean EPAP** (cmH_2_O) | | n=67  5.00  [4.30 – 6.00]  (4.00 – 11.90) | | | n=12  5.25  [4.65 – 7.05]  (4.00 – 11.00) | | n=29  4.50  [4.00 – 5.00]  (4.00 – 8.00) | | | n=13  5.40  [4.80 – 8.00]  (4.40 – 11.90) | | | n=7  6.00  [4.50– 8.20]  (4.00 – 10.00) | | | n=6  5.00  [4.00 – 5.60]  (4.00 – 6.40) | | | 0.014* | | |  |
| **Median EPAP** (cmH_2_O) | | n=89  7.20  [6.00 – 10.00]  (4.00 – 14.00) | | | n=36  8.00  [6.40 – 10.00]  (4.30 – 14.00) | | n=18  6.60  [5.00 – 8.00]  (4.00 – 13.00) | | | n=16  7.10  [5.90 – 8.60]  (4.90 – 12.00) | | | n=12  9.00  [6.50– 12.00]  (5.00 – 14.00) | | | n=7  6.00  [5.00 – 7.00]  (5.00 – 8.50) | | | 0.053 | | |  |
| **Mean 90^th^ EPAP** (cmH_2_O) | | n=67  5.20  [4.50 – 7.60]  (4.00 – 14.30) | | | n=12  6.20  [5.00 – 8.05]  (4.00 – 11.00) | | n=29 ^i^  5.00  [4.00 – 5.00]  (4.00 – 9.80) | | | n=13 ^i^  6.80  [6.00– 8.90]  (5.00 – 14.30) | | | n=7  7.40  [5.70– 9.50]  (4.00 – 10.00) | | | n=6  5.00  [4.00 – 6.70]  (4.00 – 7.00) | | | <0.001 | | |  |
| **Median 95^th^ EPAP** (cmH_2_O) | | n=90  7.95  [6.00 – 10.00]  (4.00 – 14.80) | | | n=36  8.45  [7.00 – 10.00]  (5.00 – 14.80) | | n=19  8.00  [5.00 – 9.00]  (4.00 – 13.00) | | | n=16  7.90  [6.30– 9.80]  (5.00 – 12.00) | | | n=12  7.85  [6.50– 12.00]  (4.00 – 14.00) | | | n=7  6.00  [5.00 – 7.00]  (5.00 – 9.50) | | | 0.097 | | |  |
| **Mean IPS**  (cmH_2_O) | | n=68  5.35  [1.90 – 8.20]  (0.50 – 16.40) | | | n=12 ^e^  2.65  [1.15 – 5.00]  (0.60 – 8.10) | | n=30 ^e^  7.00  [4.00 – 10.00]  (0.60 – 16.40) | | | n=13  2.70  [1.80 – 5.60]  (0.50 – 9.60) | | | n=7  6.00  [1.50 – 9.20]  (1.40 – 11.30) | | | n=6  5.50  [2.60 – 9.30]  (1.60 – 10.10) | | | 0.027 | | |  |
| **Median IPS**  (cmH_2_O) | | n=90  4.00  [3.50 – 4.60]  (1.10 – 14.10) | | | n=37  4.30  [3.60 – 4.90]  (1.10 – 9.10) | | n=19  4.20  [3.80 – 4.40]  (1.30 – 6.60) | | | n=16  3.40  [1.95 – 5.20]  (1.10 – 9.80) | | | n=11  3.90  [3.60 – 5.20]  (3.50 – 14.10) | | | n=7  3.50  [3.30 – 3.80]  (1.30 – 4.00) | | | 0.068 | | |  |
| **Mean 90^th^ IPS**  (cmH_2_O) | | n=62  7.10  [4.40 – 8.90]  (1.00 – 14.90) | | | n=12  4.60  [2.90 – 7.00]  (1.50 – 10.80) | | n=24  8.00  [6.05 – 9.00]  (1.30 – 14.90) | | | n=13  6.70  [4.90 – 7.90]  (1.00 – 14.40) | | | n=7  7.80  [3.50 – 10.80]  (3.10 – 13.80) | | | n=6  7.15  [5.70 – 11.00]  (4.10 – 12.20) | | | 0.238 | | |  |
| **Median 95^th^ IPS**  (cmH_2_O) | | n=90  7.10  [6.10 – 8.10]  (0.50 – 13.40) | | | n=37  7.10  [6.20 – 7.90]  (3.00 – 11.80) | | n=19  7.40  [6.90 – 8.20]  (2.60 – 10.30) | | | n=16  5.95  [3.75 – 9.75]  (2.80 – 13.40) | | | n=11  7.00  [6.40 – 8.70]  (0.50 – 11.90) | | | n=7  6.80  [6.30 – 8.60]  (2.40 – 9.00) | | | 0.543 | | |  |
| **Mean RR**  (cycle/min) | | n=68  15.70  [13.75 – 17.10]  (10.00 – 24.80) | | | n=12  16.85  [14.55 – 17.40]  (13.10 – 17.80) | | n=30  15.15  [13.50 – 16.70]  (11.40 – 22.30) | | | n=13  15.80  [15.10 – 17.10]  (10.00 –19.30) | | | n=7  14.90  [11.20 – 19.50]  (10.00 – 24.80) | | | n=6  14.45  [12.00 – 21.00]  (11.30 – 21.20) | | | 0.697 | | |  |
| **Median RR**  (cycle/min) | | n=108  16.00  [13.50 – 17.00]  (9.00 – 25.00) | | | n=40  16.00  [13.00 – 17.00]  (10.00 – 25.00) | | n=28  15.50  [14.00 – 17.50]  (9.00 – 19.00) | | | n=20  14.50  [13.00 – 17.00]  (11.00 –20.00) | | | n=13  16.00  [16.00 – 18.00]  (12.00 – 20.00) | | | n=7  15.00  [15.00 – 16.00]  (13.00 – 17.00) | | | 0.429 | | |  |
| **Mean**  **final AHI_flow_**  (n/h) | n=68  3.00  [2.00 – 5.45]  (0.20 – 22.60) | | | n=12  4.25  [2.95 – 7.45]  (1.00 – 18.30) | | | | n=30  2.70  [1.80 – 4.50]  (0.40 – 22.60) | | | n=13  5.50  [1.50 – 9.90]  (0.20 –17.30) | | | n=7  3.40  [2.00 – 8.20]  (1.00 – 10.80) | | | n=6  2.80  [2.40 – 4.00]  (0.70– 13.10) | | | 0.442 | | |
| **Median final AHI_flow_**  (n/h) | n=109  1.10  [0.30 – 2.80]  (0.00 – 36.50) | | | n=41  1.50  [0.30– 3.30]  (0.00 – 36.50) | | | | n=28  0.95  [0.60–2.75]  (0.00 – 17.20) | | | n=20  0.65  [0.15 – 2.30]  (0.10 –6.00) | | | n=13  1.10  [0.70 – 2.90]  (0.10 – 14.20) | | | n=7  0.80  [0.60– 2.00]  (0.40– 3.80) | | | 0.570 | | |
| **Mean**  **important leaks**  **(%)** | | n=66  1.05  [0.30 – 3.80]  (0.00 – 66.00) | | | n=12  1.90  [0.75 – 7.55]  (0.00 – 66.00) | | n=28  1.00  [0.20 – 3.20]  (0.00 – 30.00) | | | n=13  0.40  [0.20 – 0.90]  (0.00 – 14.30) | | | n=7  2.50  [0.80 – 12.10]  (0.30 – 32.30) | | | n=6  3.10  [0.00 – 5.20]  (0.00 – 5.70) | | | 0.128 | | |  |
| **Median unintentional**  **leaks (l/min)** | | n=107  0.00  [0.00 – 6.00]  (0.00 – 93.60) | | | n=40  0.60  [0.00 – 0.60]  (0.00 – 25.00) | | n=28  1.20  [0.00 – 7.20]  (0.00 – 56.40) | | | n=19  0.00  [0.00 – 1.20]  (0.00 –4.20) | | | n=13  0.00  [0.00 – 6.00]  (0.00 – 9.60) | | | n=7  0.00  [0.00 – 16.80]  (0.00 – 22.80) | | | 0.239 | | |  |
| **Mean**  **observance**  **(h/day)** | | n=68  6.41  [5.15 – 7.40]  (2.68– 11.65) | | | n=12  5.88  [4.48 – 6.43]  (2.68 – 8.12) | | n=30  6.42  [5.48 – 7.70]  (3.98 – 11.65) | | | n=13  5.93  [4.75 – 7.12]  (3.12 –9.30) | | | n=7  6.52  [4.78 – 7.90]  (4.15 – 8.65) | | | n=6  6.63  [6.52 – 8.17]  (6.35– 8.32) | | | 0.225 | | |  |
| **Median observance**  **(h/day)** | | n=109  6.55  [5.13 – 7.75]  (0.00– 13.40) | | | n=41  7.40  [4.40 – 8.32]  (0.00– 11.92) | | n=28  6.53  [5.10 –6.90]  (1.32 – 10.42) | | | n=20  6.08  [5.08 – 7.14]  (1.90–8.45) | | | n=13  6.55  [6.38 – 7.75]  (1.00 – 13.40) | | | n=7  6.33  [3.93 – 7.35]  (1.55 – 7.53) | | | 0.243 | | |  |
| **Interface** | | n=177 | n=53 | | | n=58 | | | n=33 | | | n=20 | | | n=13 | | |  | | |  |  |

Quantitative variables were summarized using medians, [IQ25-75] and (min – max), while categories were described by numbers and (%). Note that for Philips Respironics® devices, data are expressed as “mean” in the manufacturer software whereas for ResMed® device, data are expressed as “median” (thus preventing direct comparisons).

Significant (p<0.05) post-hoc pairwise comparisons after Holm correction (within lines) were presented using labels  ^e,I^. Label ^e^ indicates a significant difference between Cluster 1 and Cluster 2, label ^j^ indicates a significant difference between Cluster 2 and Cluster 3. *Pairwise comparisons using Holm correction for multiple comparison did not reached statistical significant difference.

AHI_flow_: Apnea Hypopnea Index estimated by the device, EPAP: expiratory positive airway pressure; IPS: inspiratory Pressure Support; max: maximum; min: minimum; Pmax: maximum pressure; RR: respiratory rate.
